# Canonical Wnt Signaling Drives Myopia Development and Can Be Pharmacologically Modulated

**DOI:** 10.1167/iovs.62.9.21

**Published:** 2021-07-14

**Authors:** Zhen Liu, Yanghui Xiu, Fangfang Qiu, Zhenzhen Zhu, Rongrong Zong, Xiangtian Zhou, Jianhong An, Qiongsi Wang, Peter S Reinach, Wei Li, Wensheng Chen, Zuguo Liu

**Affiliations:** 1 Eye Institute and Affiliated Xiamen Eye Center of Xiamen University, Xiamen University, Xiamen, Fujian, China; 2 Fujian Provincial Key Laboratory of Ophthalmology and Visual Science, Xiamen University, Xiamen, China; 3 School of Ophthalmology and Optometry and Eye Hospital, Wenzhou Medical University, Wenzhou, Zhejiang, China; 4 Zhejiang Provincial Key Laboratory of Ophthalmology and Optometry, Wenzhou, Zhejiang, China; 5 Center for Translational Ocular Immunology, Department of Ophthalmology, Tufts Medical Center, Tufts University School of Medicine, Boston, Massachusetts, United States; 6 Fujian Engineering and Research Center of Eye Regenerative Medicine, Xiamen University, Xiamen, Fujian, China

**Keywords:** canonical Wnt signaling pathway, DKK-1, myopia, niclosamide

## Abstract

**Purpose:**

The purpose of this study was to evaluate the role of the canonical Wnt signaling in the development of the myopia.

**Methods:**

Plasma from adult patients with myopia, myopic animal models including the adenomatous polyposis coli (APC) gene mutation mouse model, and the form deprivation (FD) induced mouse model of myopia were used. Niclosamide, a canonical Wnt pathway inhibitor, was orally administrated in animal models. Plasma levels of DKK-1 were determined by using enzyme-linked immunosorbent assay. Refraction, vitreous chamber depth (VCD), axial length (AL), and other parameters, were measured at the end of the FD treatment. Canonical Wnt signaling changes were evaluated by Western blot analysis and immunostaining analysis.

**Results:**

Plasma level of Wnt inhibitor DKK-1 was markedly decreased in patients with myopia. Meanwhile, the canonical Wnt pathway was progressively activated during myopia development in mice. Moreover, inhibition of canonical Wnt signaling by niclosamide in mouse models markedly reduced lens thickness (LT), VCD, and AL elongation, resulting in myopia inhibition.

**Conclusions:**

Dysregulation of canonical Wnt signaling is a characteristic of myopia and targeting Wnt signaling pathways has potential as a therapeutic strategy for myopia.

Myopia occurs in more than 50% of the population in many countries and is expected to increase globally.^1^ Simple myopia can be corrected with spectacles or contact lenses, whereas high myopia usually suffer from progressive elongation of the eye globe, which is accompanied by potentially blinding degenerative complications, including retinal detachment, macular degeneration, premature cataract, and glaucoma.^2^ It is generally accepted that myopia is determined by complex interactions of environmental and genetic factors.^3^ Retinal messengers pass though the retinal pigmented epithelium (RPE) and the vascular choroid to reach the fibrous sclera, which responds with axial eye growth.^4^ Animal models have shown that a number of involved messenger molecules in the retina, including dopamine (DA), acetylcholine, and ZENK, have been proposed in the development of myopia.^4^^–^^7^ However, mechanisms governing the refractive error development as well as their potential role in human myopia are not clearly elucidated.

The canonical Wnt signaling pathway is highly conserved among various species, and implicated in many biological processes during development and diseases.^8^ Wnt ligands initiate the signaling pathway by interacting with cell surface receptors Frizzled (Fz) and low-density lipoprotein receptor-related protein 5/6 (LRP5/6). Subsequently, the downstream mediators Disheveled (Dvl/Dsh) was recruited to Fz and results in disassembly of the β-catenin destruction complex consisting of Axin, adenomatous polyposis coli (APC), glycogen synthase kinase3β (GSK3β), and casein kinase 1 (CK1), ultimately leading to the nuclear accumulation of β-catenin and the activation of the canonical Wnt signaling pathway.^9^ Several human ocular pathologies are caused by mutations of known Wnt pathway genes and genes that may regulate or be affected by this pathway.^10^ Recent genome-wide association studies (GWAS) on patients with myopia also identified a high association of the Wnt pathway with myopia.^11^ Moreover, there are some experimental evidences showing that the canonical Wnt signaling pathway related proteins, including GSK-3β, Wnt7b, and Wnt2b were involved in the myopia development.^12^^–^^14^ Furthermore, inhibition of the canonical Wnt signaling pathway by antagonist Dickkopf-1 (DKK-1) reduced the myopic shift in refractive error,^13^ which suggested that canonical Wnt signaling pathway were vital for myopia development. However, the changes of canonical Wnt signaling in patients with myopia remain unexamined. In addition, activation of the canonical Wnt signaling pathway in the early stage of myopia remains elusive.

In this study, we demonstrate that myopia is associated with overactivation of Wnt signaling. We measured circulating DKK-1 levels in the patients with myopia and their association with the risk of myopia. In a form deprivation (FD) myopia mouse model that reflects the progression of myopia in humans^15^ and a nonsense mutation of APC gene (APC^Min^) mouse that developed myopia shift after birth,^16^ we find that inhibition of the canonical Wnt pathway by niclosamide results in alleviated myopia development, revealing therapeutic Wnt inhibition as a potential treatment for myopia in humans.

## Methods

### Human Subjects

A total of 197 patients with myopia and 51 age-matched healthy subjects were recruited from Xiamen Eye Center Affiliated to Xiamen University, after approval from the Human Ethics committee of the center, in accordance with the Declaration of Helsinki. Informed consents were obtained from all subjects after explanation of the nature and possible consequences of the study. All patients and healthy control subjects involved in this study were from the local ethnic Han Chinese population, with no ethnic subdivision.

All participants underwent noncycloplegic autorefraction of both eyes using the Topcon KR8800 autorefractor (Topcon, Tokyo, Japan). Patients with high myopia (<-6D) with axial lengths (AL) of 26.0 mm or more and patients with moderate myopia (−6D to approximately −0.5D) with less 26.0 mm AL were designated as study cases, whereas emmetropia control subjects (approximately 0.8D to 1.5D) with AL less 25.0 mm. Subjects with a history of intraocular surgery, ocular trauma, glaucoma, uveitis, LASIK/PRK, prophylactic laser photocoagulation, and systemic diseases, such as pseudo exfoliation, renal dysfunction, hepatic dysfunction, diabetes mellitus, ischemic cerebrovascular disorders, ischemic cardiovascular disorder, hematological diseases, connective tissue and systemic inflammatory diseases, and history of malignancy, bone diseases, neurological disorder, and antiplatelet therapy were excluded from the study.

### Collection of Blood Samples and Measurement of DKK-1 Levels

Blood samples, drawn from the antecubital vein, were collected into tubes containing heparin. The samples were centrifuged at 3000 RPM for 10 minutes at 4°C, and the plasma was separated and stored at −80°C within 2 hours for further analysis. DKK-1 levels in clinical plasma samples were measured using a commercially available enzyme-linked immunosorbent assay (ELISA) kits, according to the recommendations of the manufacturer (R&D Systems, Minneapolis, MN, USA). All measurements were performed in triplicate for each sample, and the mean value was calculated. Inter- and intra-assay variations were 3.1% and 5.6%, respectively.

### Animals

APC^Min^ mice (stock number 002020; The Jackson Laboratory) on the C57BL/6 background and wild-type (WT) C57BL/6 mice (purchased from Shanghai SLAC Laboratory Animal Center, Shanghai, China) were used, treated and cared for in accordance with the ARVO Statement for the Use of Animals in Ophthalmic and Vision Research and the Guidelines of the Animal Experimental Committee of Xiamen University. All animals were housed in units at 25°C, at 12:12 light-dark hours, with food and water were available ad libitum. APC^Min^ mice developed adult-onset anemia and multiple intestinal neoplasia (Min). The genotype of the mice was determined following a PCR protocol recommended by The Jackson Laboratory.

### Form-Deprivation Treatment and Biometric Measurements

FD as produced by covering a translucent diffuser to the right eye (OD) of the animals for 1 day, 7 days, and 28 days, and the lateral eye (OS) was maintained free from FD. The hand-made diffuser was attached carefully to the fur around one eye on postnatal day 30 with cyanoacrylic glue during ether anesthesia.^17^ Biometric measurements, including corneal radius of curvature (CRC), refractive error, anterior chamber depth (ACD), lens thickness (LT), vitreous chamber depth (VCD), and axial length (AL) has been described previously.^18^ Briefly, refractive error of the eye was measured using an eccentric infrared photorefractor without anesthesia. The CRC was measured with a keratometer (OM-4; Topcon Corporation, Dongguan, Japan), which was modified by mounting a +20.0 diopter (D) aspherical lens; ACD, LT, VCD, and AL were measured by real-time optical coherence tomography (OCT; a custom-made OCT). ACD was defined as the distance from the posterior surface of the cornea to the anterior surface of the lens. AL was defined as the distance between the anterior surface of the cornea and the vitreous-retina interface. Each eye was measured three times to obtain a mean value.

### Drug Administration

Four-week-old C57BL/6 male mice (weighting 13–15 g) and 10 weeks old APC^Min^ mice received once-daily oral administration of 100 µL of 40 mg/mL niclosamide (200 mg/kg of body weight; Sigma) by gavage as the niclosamide group, whereas the other mice were treated with PBS by gavage as the control group. After 4 weeks of treatment, the mice were euthanized 24 hours after the last drug administration, and retina tissues were collected.

### Immunofluorescence

Cryostat sections (10 µm in thickness) of each eyeball were fixed in cold acetone, and blocked with 2% normal bovine serum for 1 hour at room temperature. Sections were incubated overnight with primary antibodies specific for β-catenin (Santa Cruz; catalog no. sc-7199, 1:400), Dvl3 (Cell Signaling Technology; catalog no. # 3218T, 1:1000), APC (Santa Cruz; catalog no. sc-896, 1:400), and washed thoroughly with phosphate-buffered saline. After further incubation with species-appropriate secondary antibodies conjugated to FTIC-488 (Thermo Fisher Scientific; catalog no. A-21206, 1:1000), sections were counterstained with DAPI, mounted, and photographed using a confocal laser scanning microscope (Fluoview 1000; Olympus, Tokyo, Japan).

### Western Blot Analysis

Proteins of the retina from each group were extracted with cold radio-immunoprecipitation assay (RIPA) buffer containing 25 mM Tris•HCl pH 7.6, 150 mM NaCl, 1% NP-40, 1% sodium deoxycholate, 0.1% SDS, and a proteinase inhibitor cocktail (catalog no. 78440; Thermo Fisher Scientific, Waltham, MA, USA). Tissue lysates were centrifuged at 15,000 rpm for 10 minutes at 4°C, and quantitate the total protein by the BCA protein Assay Kit (Pierce; 23225). After boiling in SDS-PAGE buffer, samples were separated by 8% SDS-PAGE and the proteins were transferred onto a polyvinylidene difluoride membrane (Millipore, Inc.). Membranes were blocked with 2% bovine serum for 1 hour at room temperature and then incubated overnight at 4°C with phospho-β-catenin (Ser33/37/Thr41; p-β-catenin; Cell Signaling Technology; catalog no. #9561, 1:1000), β-catenin (Santa Cruz; catalog no. sc-7199, 1:400), GSK-3β (Cell Signaling Technology; catalog no. # 9315S, 1:1000), phosphorylated GSK-3β (Cell Signaling Technology; catalog no. #9331S, 1:1000), Dvl3 (Cell Signaling Technology; catalog no. # 3218T, 1:1000), and APC (Santa Cruz; catalog no. sc-896, 1:500) primary antibodies. Detection was achieved with anti-rabbit-horseradish peroxidase (R&D Systems Inc., Minneapolis, MN, USA; 1:5000, catalog no. HAF008) and ECL Western blotting detection agents (catalog no. ECL-500; Lulong, Inc., Xiamen, China). Each protein band was normalized by β-actin levels in the same gel.

### Statistical Analysis

Groups were compared with either a 2-tailed Student's *t*-test (for analysis of 2 groups) or using 1-way ANOVA to compare multiple groups. Statistical significance was set at *P* < 0.05.

## Results

### Plasma Level of Wnt Inhibitor DKK-1 was Markedly Decreased in Patients With Myopia

**Figure 1.**
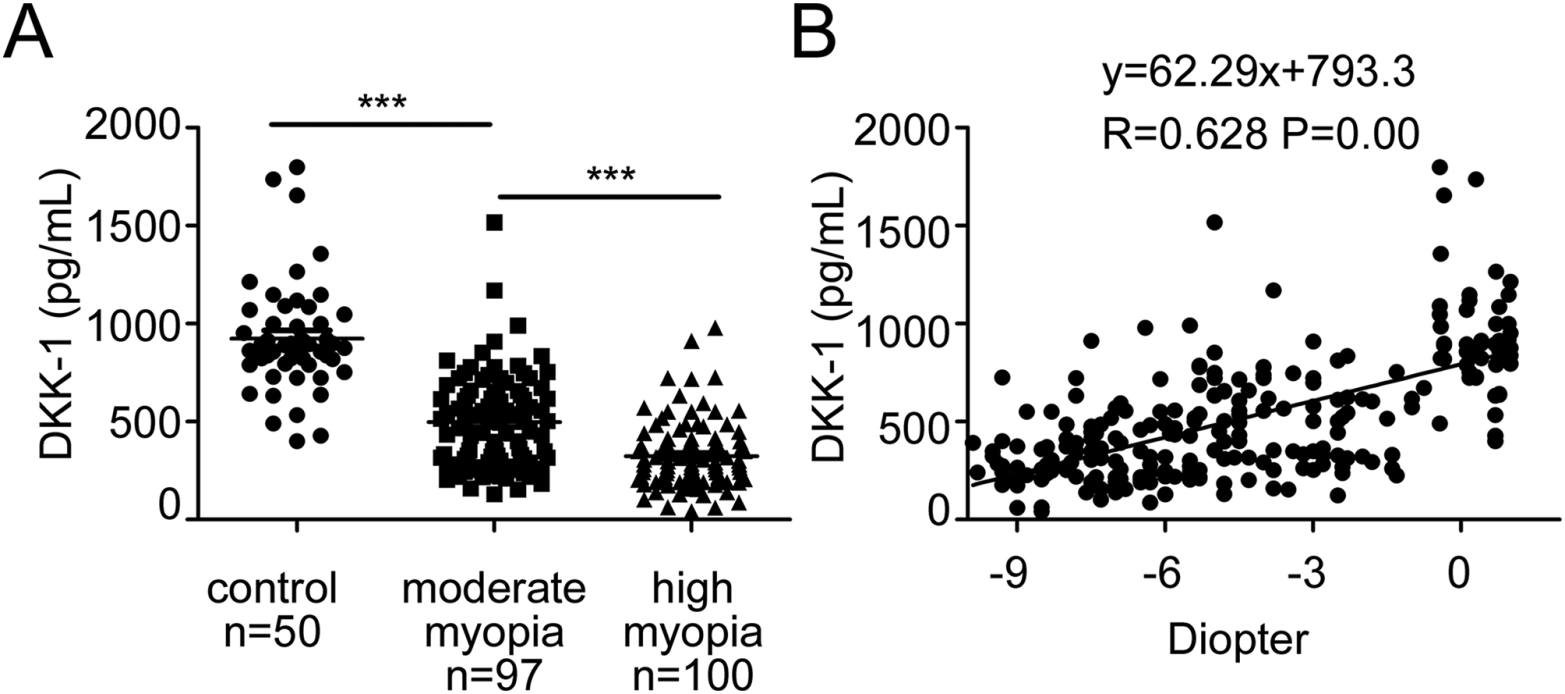
**Decreased levels of plasma DKK-1 were associated with severity of human myopia.** (**A**) Plasma DKK-1 levels in the high myopia patients were lower compared with the normal control group and moderate myopia patients. one-way ANOVA, Data are presented as mean ± SEM, ***P < 0.001. (**B**) Linear regression relationships among DKK-1 and diopter were plotted using Pearson correlation coefficient.

DKK-1 is a soluble inhibitor of the canonical Wnt signaling pathway, and platelets are the major producer of circulating DKK-1.^19^ Accurate and precise measurements of circulating DKK-1 would be useful for the clinical investigation of the abnormalities of Wnt signaling in pathological situations.^20^ In our study, we measured the plasma levels of DKK-1 in patients with myopia and healthy controls. The clinical characteristics of the subjects are summarized in Table 1. Plasma samples were collected from 197 patients with myopia, including 100 patients with high myopia (<-6D) and 97 patients with moderate myopia (approximately −6D to −0.5D) and, and 50 non-myopic controls. There was a statistically significant difference of DKK-1 levels in the plasma among the three groups (*P* < 0.001). As shown in Figure 1A, the levels of DKK-1 were significantly lower in the high myopia group (median = 324.25 pg/mL, range = 42.2–978.79 pg/mL) in comparison with those in moderate myopia groups (496.67 pg/mL, 130.23–1517.74 pg/mL; *P* < 0.001, 1-way ANOVA) and non-myopic controls (924.86 pg/mL, 400.08–1799.36 pg/mL; *P* < 0.001, 1-way ANOVA). Furthermore, there was a positive relationship between DKK-1 concentration and refraction error (diopter; R = 0.628, *P* = 0.003; Fig. 1B). These findings demonstrated that decreased DKK-1 levels in the circulation are associated with the presence or development of myopia.

**Table 1.** Clinical Characteristics of Subjects and Distribution of DKK-1 Levels

Characteristic	Healthy Control *n* = 51	Moderate Myopia *n* = 97	High Myopia *n* = 100
Sex, *n* (%)			
Female	27 (52.9)	71 (73.2)	49 (49)
Male	24 (47.1)	26 (26.8)	51 (51)
Age (years)			
Mean ± SD	21.88 ± 1.96	23.17 ± 4.67	22.64 ± 5.89
Median (range)	22 (18–26)	22 (17–38)	20 (17–42)
DKK-1 (pg/mL)			
Mean ± SD	988.39 ± 534.33	496.67 ± 237.56	324.25 ± 171.82
Minimum	400.08	130.23	42.20
Maximum	1799.36	1517.74	978.79
Axial length (mm)			
Mean ± SD	24.37 ± 0.51	24.82 ± 0.88	27.35 ± 1.40
Minimum	23.17	22.10	26.00
Maximum	25.06	26.02	32.22
Refractive diopter (D)			
Mean ± SD	1.186 ± 0.24	−3.682 ± 1.41	−7.5 ± 2.73
Minimum	0.80	−5.75	−19.50
Maximum	2.00	−0.75	−6.00

### The Canonical Wnt Pathway is Progressively Activated During Myopia Development

Because human plasma samples reflect systemic changes of the canonical Wnt pathway, experiments using such samples do not address whether the Wnt pathway activation in the retina is a hallmark of myopia development. To evaluate the activation status of the canonical Wnt pathway in the myopia, we used a mouse model.

**Figure 2.**
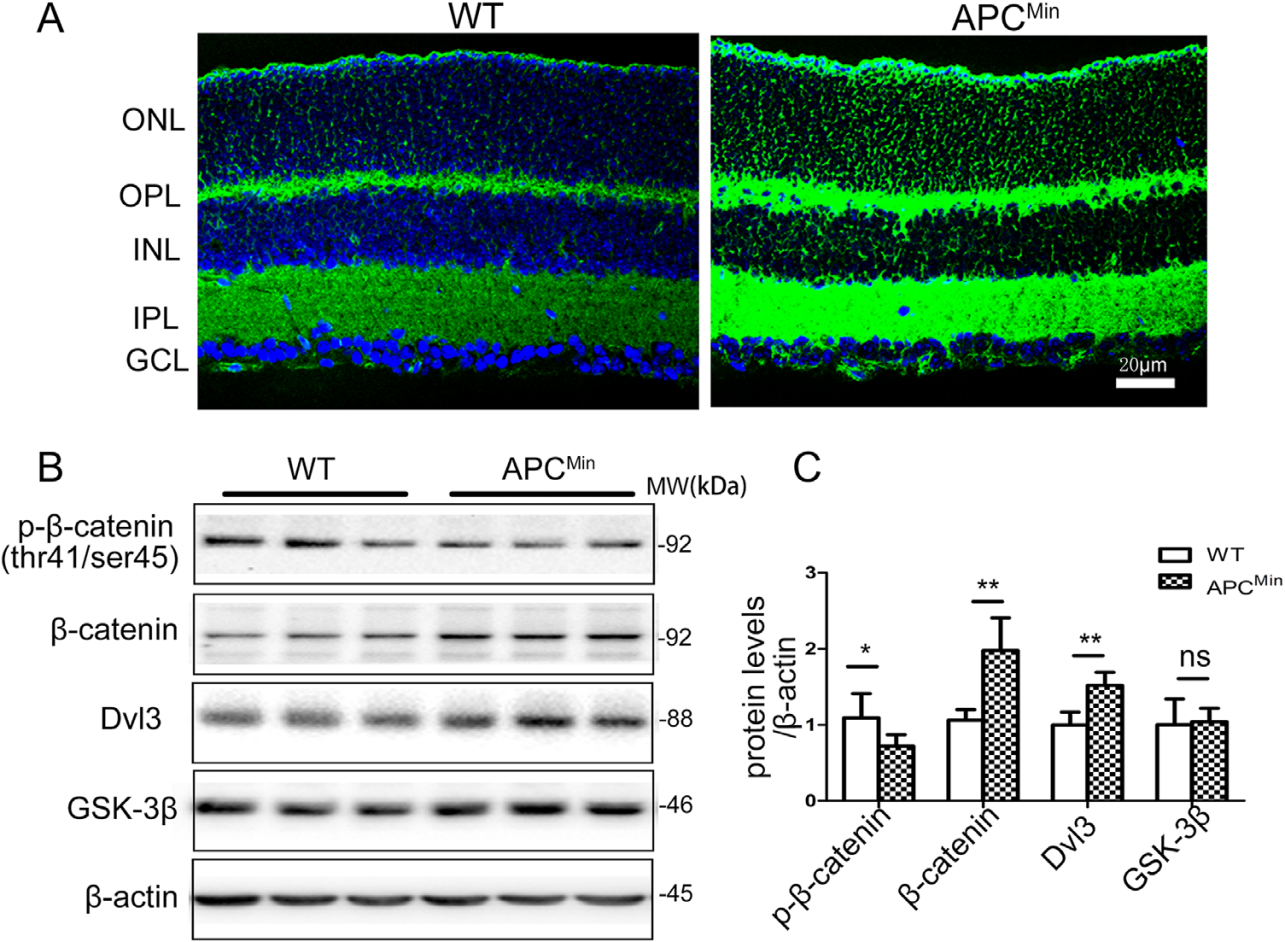
**Activation of the canonical Wnt signaling pathway in APC^Min^ mice.** (**A**) Ocular sections from wt and APC^Min^ mice on P84 were stained with the anti-β-catenin antibody (*green*), and the nuclei were counterstained by DAPI (*blue*). (**B**) The same amount of eyecup proteins (100 µg) was blotted separately with antibodies for phosphorylated-β-catenin, total β-catenin, Dvl3, and GSK3β. (**C**) Semi quantified by densitometry and normalized by β-actin levels. The Figure is a summary of two independent experiments with *n* = 6 eyes per group Student *t*-test. Data are presented as mean ± SEM, *P < 0.05, **P < 0.01. ns, no difference.

We have recently described the APC^Min^ mouse model of myopia in which a nonsense mutation of APC gene at codon 850 was introduced using random ethylnitrosourea (ENU) mutagenesis.^16^ To determine whether the canonical Wnt signaling pathway was activated in the retina of APC^Min^ mouse, some relevant proteins were examined. Western blot analysis showed that significant upregulation of β-catenin and Dvl3 in the retina compared with their WT control mice, whereas GSK3β protein level was unchanged (Fig. 2B). Consistent with this finding, the mutation of APC greatly inhibited phosphorylation of β-catenin at Thr41 (see Fig. 2B). We also immunostained total β-catenin in the retina section of the APC^Min^ mouse and could detect staining in the membrane, but also strong staining in the cytoplasm and nucleus of the photoreceptor cells (Fig. 2A). These data indicate activation of the canonical Wnt signaling pathway by the mutation of the APC gene.

**Figure 3.**
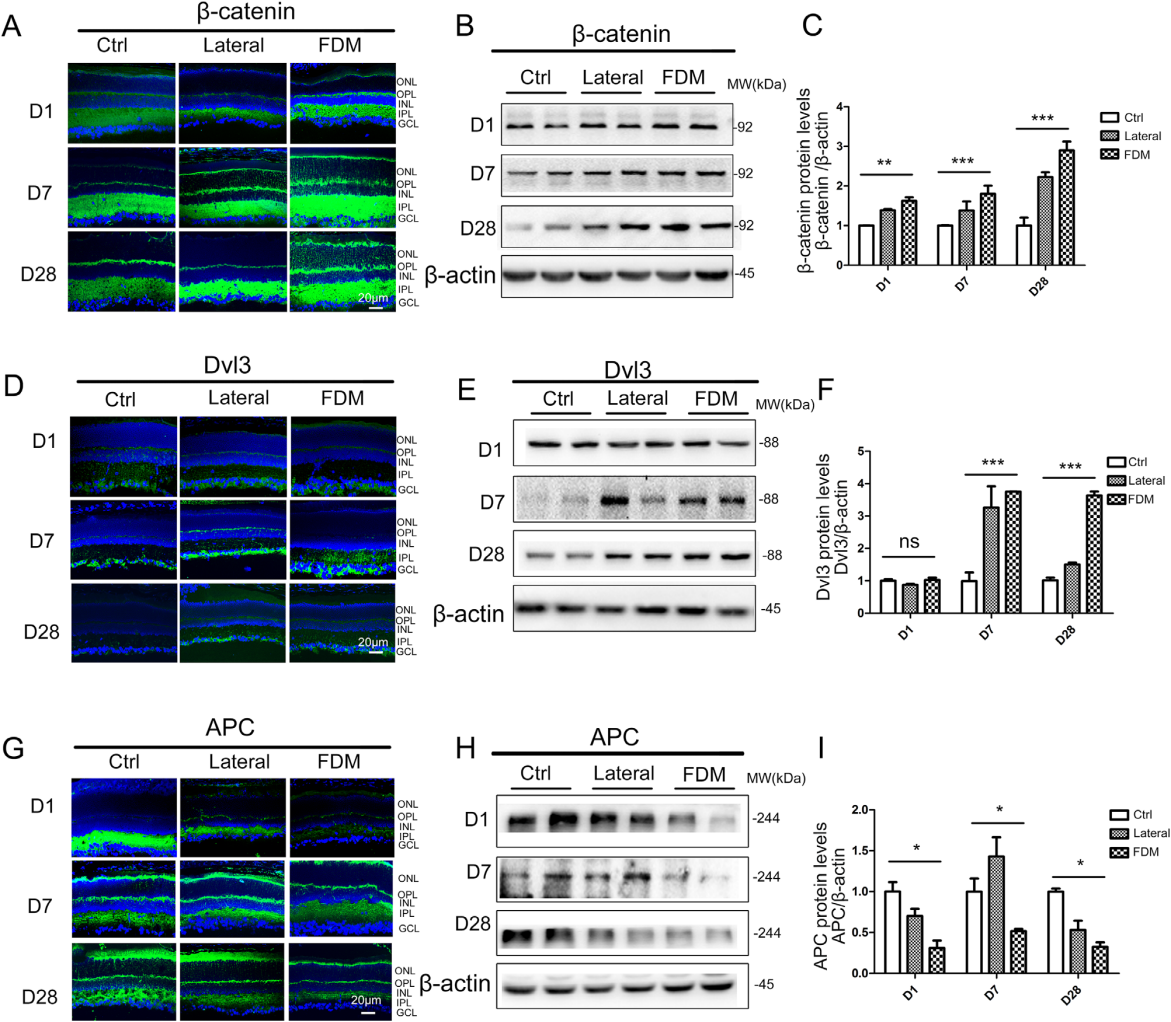
**The canonical Wnt pathway is progressively activated during myopia development.** (**A,** **B**) β-catenin expression was evaluated by immunostaining and western blot analysis in FDM and normal control groups following day 1, day 7, and day 28 of treatment. (**C**) β-catenin protein levels were semi quantified with densitometry and normalized by β-actin levels. (**D,** **E**) Dvl3 expression was evaluated by immunostaining and Western blot analysis in FDM and normal control groups following day 1, day 7, and day 28 of treatment. (**F**) Dvl3 protein levels were semi quantified with densitometry and normalized by β-actin levels. (**G,** **H**) APC expression was evaluated by immunostaining and Western blot analysis in FDM and normal control groups following day 1, day 7, and day 28 of treatment. (**I**) APC protein levels were semi quantified with densitometry and normalized by β-actin levels. Data are presented as mean ± SEM, *n* = 10, *P < 0.05, **P < 0.01, compared with the normal group and lateral eye of FDM.

To assess experimentally how the canonical Wnt pathway regulates myopia development without prior genetic modification, we used FD in mice as a more appropriate model of myopia. FD induces elongation of AL, culminating in myopia development after 4 weeks in the mouse that resembles the human myopia.^21^ Throughout the early stages of the FD model of myopia (from day 1), the canonical Wnt pathway was highly activated in FD-treated versus non-FD age-matched controls. Western blot analysis showed that β-catenin was significantly higher in the FD myopia eyes than in the lateral eyes and control eyes (*P* < 0.05, 1-way ANOVA) after treatment for 1, 7, and 28 days (Figs. 3B, 3C). We also stained for total β-catenin in the retina and could detect staining in the membrane, but also strong staining in the cytoplasm and nucleus, indicating that the canonical Wnt signaling pathway is active (Fig. 3A). To further assess the activation status of the canonical Wnt pathway, retinal protein levels of Dvl3 and APC were measured by immunostaining and Western blot. The expression of Dvl3 was significantly increased in the retina with FD myopia than that in the lateral eyes and that in control animals (*P* < 0.05, 1-way ANOVA) after treatment for 7 and 28 days (Figs. 3D, 3E, 3F). Meanwhile, Western blot and immunostaining analysis showed that protein levels of APC were significantly lower in the retina of FD mice, compared with those in normal control mice (Figs. 3G, 3H, 3I).

**Figure 4.**
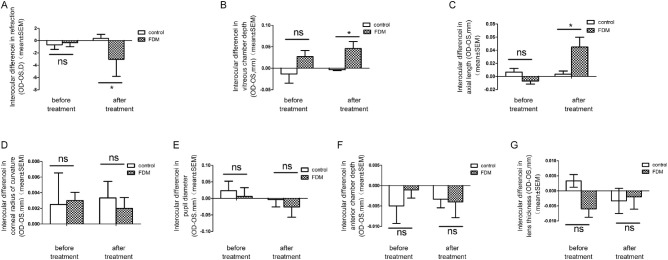
**Eyes treated with FDM showed a significantly lower myopia (interocular difference).** (**A**) With a longer vitreous chamber depth (**B**) and axial length (**C**), compared with the normal control groups Results in corneal radius of curvature (**D**), pupil diameter (**E**), ACD (**F**), and LT (**G**) were not affected by FDM treatment (data are presented as mean ± SEM, *P < 0.05, Student *t*-test).

There was no significant difference in the eyes between the two groups for all baseline. The *P* value was *P* > 0.05 for all by Student *t*-test (Table 2). The inter-ocular difference in refraction for the FD group was −3.04 ± 2.77 D compared with 0.36 ± 0.65 D for the control group (*P* < 0.05, *t*-test) at week 4 of the experiment, with an increased inter-ocular difference in AL (0.046 ± 0.07 mm in FD versus 0.005 ± 0.008 mm in controls, *P* < 0.05, *t*-test) and VCD (0.045 ± 0.07 mm in FD versus 0.005 ± 0.007 mm in controls, *P* < 0.05, *t*-test; Table 3, Figs. 4A, 4B, 4C). No significant inter-ocular difference was observed in corneal radius of curvature, pupil diameter, anterior chamber depth, and LT between the FD group and control group (*P* > 0.05; see Table 3, Figs. 4D, 4E, 4F, 4G) at week 4 of the experiment.

**Table 2.** Baseline Measurements of Refraction, and Ocular Biometry (Mice Aged 4 Weeks)

Group	Eye	Refraction (D)	CRC (mm)	ACD (mm)	LT (mm)	VCD (mm)	AL (mm)
Control *n* = 6	OS	−2.84 ± 1.34	1.34 ± 0.01	0.33 ± 0.07	1.45 ± 0.02	0.73 ± 0.02	1.90 ± 0.02
	OD	−3.52 ± 0.66	1.41 ± 0.01	0.32 ± 0.04	1.46 ± 0.02	0.72 ± 0.05	1.90 ± 0.03
FDM *n* = 20	OS	−2.02 ± 1.65	1.34 ± 0.09	0.32 ± 0.01	1.45 ± 0.02	0.69 ± 0.06	1.89 ± 0.03
	OD	−2.34 ± 1.65	1.35 ± 0.07	0.32 ± 0.01	1.45 ± 0.02	0.72 ± 0.02	1.88 ± 0.03

Data are expressed as the mean ± SEM.

CRC, corneal radius of curvature. ACD, anterior chamber depth; LT, lens thickness; VCD, vitreous chamber depth; AL, axial length; FDM, form deprivation myopia; OS, left eye; OD, right eye.

**Table 3.** Refraction and Ocular Biometry at Week 4 of FDM in Mice (Aged 8 Weeks)

Group	Eye	Refraction (D)	CRC (mm)	ACD (mm)	LT (mm)	VCD (mm)	AL (mm)
Control *n* = 6	OS	0.04 ± 0.50	1.39 ± 0.08	0.40 ± 0.02	1.68 ± 0.01	0.66 ± 0.01	2.85 ± 0.02
	OD	0.4 ± 1.1	1.42 ± 0.06	0.39 ± 0.01	1.67 ± 0.02	0.65 ± 0.03	2.85 ± 0.01
FDM *n* = 20	OS	0.69 ± 2.53	1.41 ± 0.03	0.41 ± 0.01	1.66 ± 0.02	0.66 ± 0.07	2.81 ± 0.07
	OD	−2.35 ± 2.31^*^	1.43 ± 0.01	0.41 ± 0.01	1.65 ± 0.02	0.70 ± 0.03^*^	2.86 ± 0.06^*^

Data are expressed as the mean ± SEM.

CRC, corneal radius of curvature. ACD, anterior chamber depth; LT, lens thickness; VCD, vitreous chamber depth; AL, axial length. FDM, form deprivation myopia; OS, left eye; OD, right eye.

* *P* < 0.05 compared with the control and FDM groups, independent *t*-test.

Together, these data suggest a persistent activation of the canonical Wnt signaling pathway in the retina during FD myopia in mice.

### Inhibition of Canonical WNT Signaling Attenuates Myopia Progression

**Figure 5.**
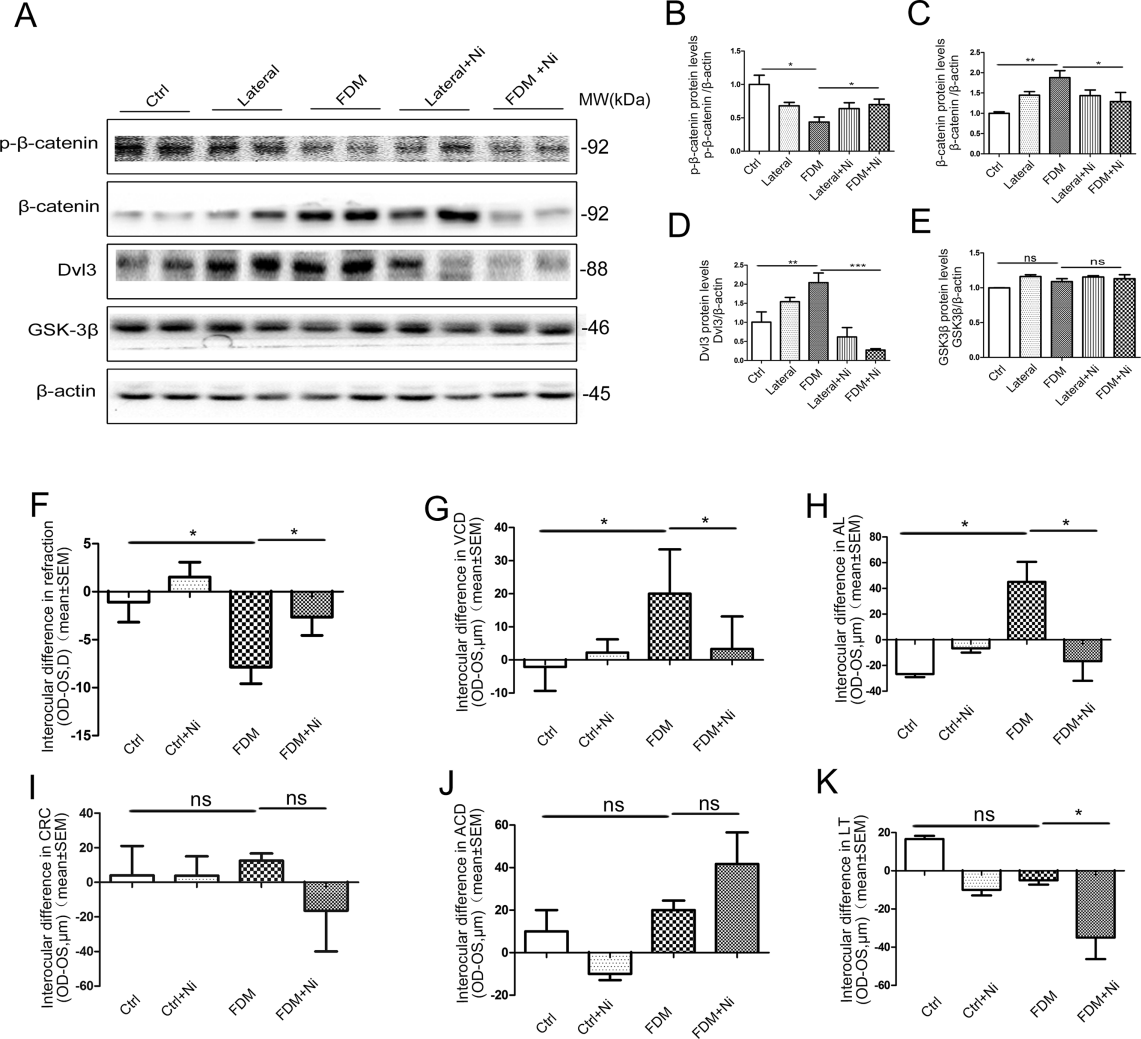
**Inhibition of the canonical Wnt signaling pathway by niclosamide reduces FDM in mice.** (**A**) The p-β-catenin, β-catenin, Dvl3, and GSK3β levels were measured by Western blot analysis using the antibodies. p-β-catenin (**B**), β-catenin (**C**), Dvl3 (**D**), and GSK3β (**E**) protein levels were semi quantified by densitometry, normalized by β-actin levels, and expressed as relative ratios normal control group, FD group, and FD group treated with niclosamide. Normal control *n* = 6, FD *n* = 10, FD + niclosamide *n* = 10. *P < 0.05, **P < 0.01, ***P < 0.001. ns, no difference; 1-way ANOVA. Eyes treated with FD-niclosamide showed a significantly lower myopia (interocular difference) (**F**), with a shorter VCD (**G**), AL (**H**), and a smaller LT (**K**) compared with the FD group. There was no difference in the CRC (**I**) and ACD (**J**) among the four groups. Data are presented as mean ± SEM, *P < 0.05, **P < 0.01, ***P < 0.001, 1-way ANOVA.

To further confirm whether the canonical Wnt pathway is required for myopia development, we administered a canonical Wnt signaling inhibitor to mice treated with FD. Our results showed that Dvl-3 was highly upregulated in the FD myopia (see Figs. 3D, 3E), we specifically inhibited the activity of Dvl-3 in FD myopia mice in vivo with oral gavage of a small molecule niclosamide, which is a US Food and Drug Administration (FDA)-approved anthelminthic drug that has been previously identified as a canonical Wnt pathway inhibitor.^22^ At day 28 of niclosamide treatment for FD mice, we observed upregulation of p-β-catenin, and large-scale downregulation of total β-catenin and Dvl-3 levels, compared with the untreated FD-only group (Fig. 5A). The results suggested that the activation of canonical Wnt pathway was inhibited by the niclosamide in the retina of the FD mice.

Subsequently, inhibition of the canonical Wnt pathway by niclosamide resulted in a marked decrease in myopia development (Fig. 5F). Moreover, the interocular difference of VCD and AL in the FD-niclosamide group were significantly decreased compared with FD-only group (Figs. 5G, 5H). Further, we also found that in the FD-niclosamide group, the interocular difference in ACD was increased (Fig. 5J), whereas the interocular difference in LT was decreased, compared to the FD-only group (Fig. 5K). There was no difference in the CRC among all the groups (Fig. 5I), indicating that canonical Wnt inhibition by niclosamide modulates the changes of ACD, LT, VCD, and AL but does not affect the growth of CRC.

**Figure 6.**
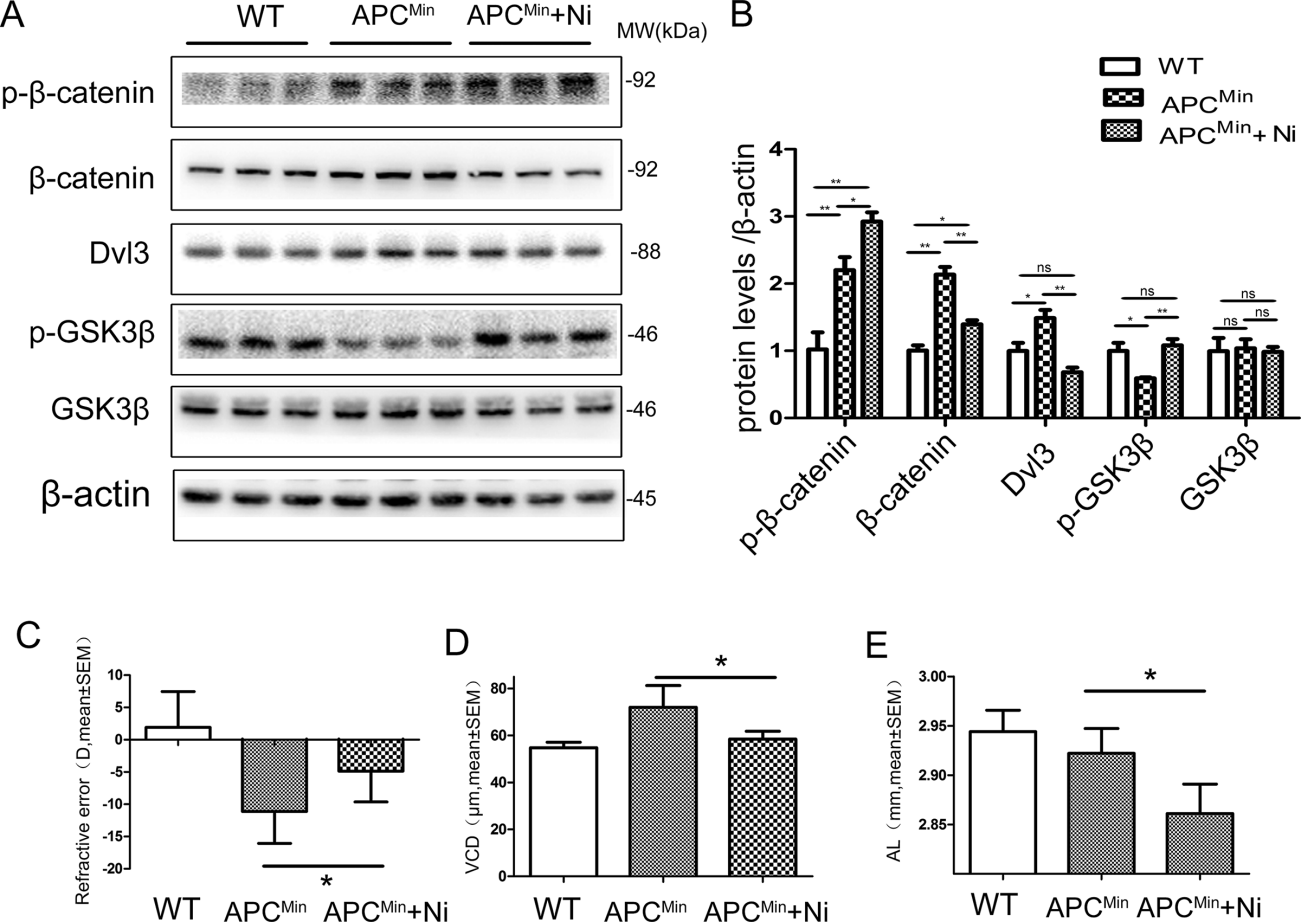
**Inhibition of the canonical Wnt signaling pathway by niclosamide reduces myopia development in APC^Min^ mice.** (**A**) The p-β-catenin, β-catenin, Dvl3, p-GSK3β, and GSK3β levels were measured by Western blot analysis. (**B**) The p-β-catenin, β-catenin, Dvl3, p-GSK3β, and GSK3β were semiquantified by densitometry, normalized by β-actin levels, and expressed as relative ratios normal control group, APC^Min^ group, and APC^Min^ group treated with niclosamide. Eyes of APC^Min^ mice treated with niclosamide showed a significantly lower myopia (**C**), with a smaller VCD (**D**) and AL (**E**), compared with the wild type group and APC^Min^ group. Data are presented as mean ± SEM, *P < 0.05, **P < 0.01, ***P < 0.001. ns, no difference; 1-way ANOVA, *n* = 10.

To further test whether niclosamide is efficacious in reducing myopia development, the 10-week-old APC^Min^ mice administered by gavage with niclosamide, and the same amounts of distilled water for control. We found a reduction of total β-catenin protein levels in the retina with the niclosamide treatment. In line with this, we observed a reduction of Dvl3 and an increment of p-β-catenin (Figs. 6A, 6B). Concurrently, we found a decrease in myopia development reflected in shorter VCD and AL within the niclosamide, compared with age-matched control mice (Figs. 6C, 6D, 6E).

In this study, neither niclosamide-treated animals demonstrated any additional chronic disease. Weight monitoring and body condition were evaluated during these studies, and no differences were detected.

## Discussion

**Figure 7.**
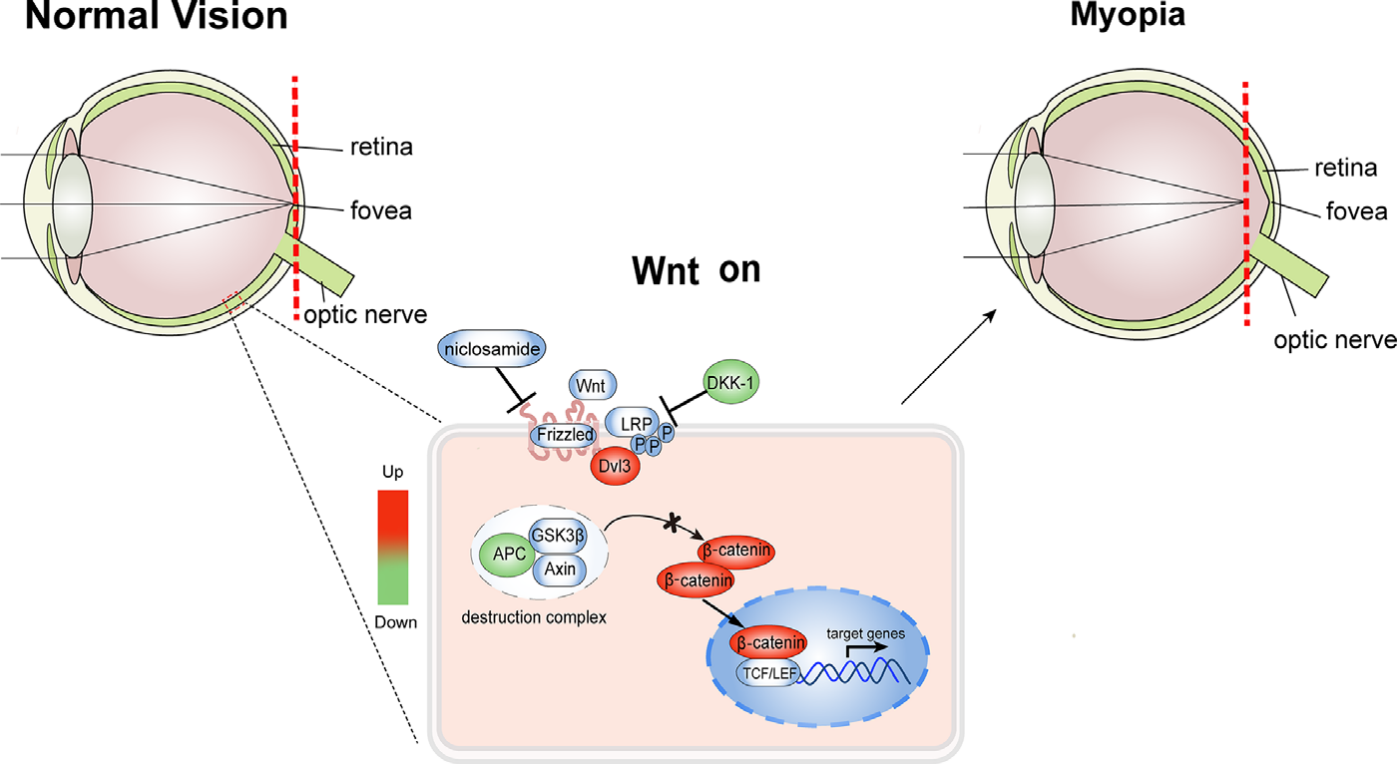
**Schematic diagram of canonical Wnt pathway activated in retina of FDM mice.** Dvl-3 increased and APC decreased in the FDM mice, which lead to the nuclear accumulation of β-catenin and activation of canonical Wnt signaling pathway. Meanwhile, inhibition of the canonical Wnt pathway by niclosamide or DKK-1 results in alleviated myopia development.

The present study demonstrated a link between refractive development and canonical Wnt activation in human myopia and mouse model of myopia (Fig. 7). More importantly, we clearly show that inhibition of the canonical Wnt pathway through niclosamide in vivo significantly reduces both AL and myopia shift, revealing niclosamide as a potential treatment for myopia.

Understanding how the canonical Wnt signaling cascade is regulated in a complex cellular environment will be important for the treatment of Wnt-dependent diseases. Studies have demonstrated that expression of many canonical Wnt signal-related genes can be altered, which cause the activation of Wnt signal in myopi.^13^^,^^14^^,^^23^ Here, our study also revealed the activation of the canonical Wnt signaling pathway. This is the first study reporting that a rapid-onset and long-lasting increased expression of β-catenin and decreased expression of APC in form deprivation myopia (FDM) mice. The greatly increased expression of β-catenin occurs prior to the onset of myopia development and persists with the ongoing myopia. This is consistent with previous studies that the GSK-3β mRNA was decreased as determined by microarray analysis after 4 hours of FD treatment.^12^ Therefore, our results support the idea that the canonical Wnt signaling pathway may be among the important pathway that are activated by form-deprivation-induced myopia, and is thus involved in myopia development. Moreover, the transcription of canonical Wnt target genes, such as matrix metalloproteinase (MMP) MMP-2, MMP-3, MMP-7 and MMP-9,^24^ is mediated after stabilization and translocation of β-catenin to the nucleus,^25^ and some of which were involved in the myopia through regulation of scleral collagen metabolism.^26^ Thus, further investigation needs to be performed to identify whether the canonical Wnt signaling-regulated MMPs cascade is a critical determinant for the development of myopia.

DKK-1 as a Wnt inhibitor plays important roles in the canonical Wnt signaling regulatory processes. It has been shown that the circulating DKK-1 levels were decreased in patients with gastric cancer, colorectal cancer, ovarian cancer, and cervical adenocarcinoma.^27^ Our previous study also found that decreased plasma DKK-1 levels are associated with the progression of diabetic retinopathy (DR),^28^ in which activation of the Wnt pathway plays a pathogenic role. Interestingly, we also found that DKK-1 levels in the plasma are significantly decreased in patients with myopia but not in normal control, and that the reduction of DKK-1 is also associated with the severity of myopia, which may provide evidence supporting an association of overactivation of the canonical Wnt signaling pathway and human myopia. Nonetheless, a report from Zhang noted raised concentrations of DKK-1 in vitreous fluid of patients with pathological myopia, which is in disagreement with our findings in plasma.^29^ Vitreous fluid obtained from patients with myopia could be a useful material for indirectly exploring special factor levels in retina. However, blood–retinal barrier (BRB) permeability changed abnormally with the development of myopia, which tend to leak more substances from the blood to the retina and less substances from the retina into the blood, compared with emmetropic eyes.^30^^,^^31^ Thus, the abnormality of the BRB that exists in myopia could affect the vitreous level of DKK-1, which may need to be further investigated after normalization to total vitreous protein concentrations. Meanwhile, larger prospective trials should be performed to further validate whether the patients with myopia with low levels of DKK-1 prior to any therapy had a significantly more myopic development than those patients with high DKK-1 levels. However, DKK-1 is not specific to the canonical Wnt signaling pathway because it has strongly crosstalk with other signaling pathways independently, such as p53 signaling^32^ and Jun amino-terminal kinase (JNK).^33^ Hence, the levels of some other inhibitors of the canonical Wnt signaling pathway in the myopia patient's plasma and local retina should be detected in the future.

Niclosamide, as one of Wnt signaling pathway inhibitors, was approved by the FDA for use in humans to treat tapeworm infections in 1982, and currently listed on the World Health Organization's (WHO's) list of essential medicines.^34^ Previous reports have shown that it downregulated canonical Wnt signaling and conferred antitumor effects of niclosamide in vitro and in vivo.^35^ In our study, we also found that orally administered niclosamide inhibited the canonical Wnt signaling pathway activation, downregulated Dvl3, decreased downstream β-catenin signaling, and exerted anti-extension of AL and VCD in the FD myopia mice and APC^Min^ mice. Thus, the pathological changes presented in myopia mouse models were attenuated by niclosamide treatment in this study, indicating that inactivation of the canonical Wnt signaling pathway inhibited the eye growth associated with myopia pathogenesis. The same beneficial effects of niclosamide could be expected on the patients with myopia, although the safety remains to be further studied.

In summary, we demonstrate an association between disease progression and canonical Wnt signaling pathway activation in human myopia and mouse model of myopia, which provided convincing evidence that inhibition of canonical Wnt is an effective treatment for myopia. Based on our findings, further investigation is required to elucidate whether the canonical Wnt signaling pathway upregulation led to the abnormal metabolism of scleral collagen in the development of myopia. We believe that understanding of the regulatory role of canonical Wnt signaling in myopia progression would facilitate the development of targeted anti myopia therapies.

### Study Approval

All animal experiments were performed in accordance with the ethics approval obtained from the Animal Experimental Committee of Xiamen University. For human samples, all blood samples were acquired from Xiamen Eye Center Affiliated to Xiamen University. Informed consent for research was given by patients for all samples. The procedures related to human subjects were approved by Ethic Committee of the Institutes of Medical School, Xiamen University.
